# Lipid signatures of chronic pain in female adolescents with and without obesity

**DOI:** 10.1186/s12944-022-01690-2

**Published:** 2022-08-30

**Authors:** Paula A. Gonzalez, Judith Simcox, Hershel Raff, Gina Wade, Helaina Von Bank, Steven Weisman, Keri Hainsworth

**Affiliations:** 1 Department of Biochemistry, University of Wisconsin-Madison, Madison, WI USA; 2 Departments of Medicine (Endocrinology and Molecular Medicine), Surgery, and Physiology, Medical College of Wisconsin, Milwaukee, WI USA; 3 Endocrine Research Laboratory, Aurora St. Luke’s Medical Center, Advocate Aurora Research Institute, Milwaukee, WI USA; 4 Departments of Anesthesiology and Pediatrics, Medical College of Wisconsin, Milwaukee, WI USA; 5 Jane B. Pettit Pain and Headache Center, Children’s Wisconsin, Wauwatosa, WI 53226 USA; 6 Department of Anesthesiology, Medical College of Wisconsin, Milwaukee, WI USA

**Keywords:** Pediatric, Lipidomics, Inflammation, Ceramide, Sphingomyelin, Plasmalogen

## Abstract

**Background:**

Chronic pain in adolescence is associated with diminished outcomes, lower socioeconomic status in later life, and decreased family well-being. Approximately one third of adolescents with chronic pain have obesity compared to the general population. In obesity, lipid signals regulate insulin sensitivity, satiety, and pain sensation. We determined whether there is a distinct lipid signature associated with chronic pain and its co-occurrence with obesity in adolescents.

**Methods:**

We performed global lipidomics in serum samples from female adolescents (*N* = 67, 13–17 years old) with no pain/healthy weight (Controls), chronic pain/healthy weight (Pain Non-obese), no pain/obesity (Obese), or chronic pain/obesity (Pain Obese).

**Results:**

The Pain Non-obese group had lipid profiles similar to the Obese and Pain Obese groups. The major difference in these lipids included decreased lysophosphatidylinositol (LPI), lysophosphatidylcholine (LPC), and lysophosphatidylethanolamine (LPE) in the three clinical groups compared to the Control group. Furthermore, ceramides and sphingomyelin were higher in the groups with obesity when compared to the groups with healthy weight, while plasmalogens were elevated in the Pain Obese group only.

**Conclusions:**

Serum lipid markers are associated with chronic pain and suggest that specific lipid metabolites may be a signaling mechanism for inflammation associated with co-occurring chronic pain and obesity.

**Supplementary Information:**

The online version contains supplementary material available at 10.1186/s12944-022-01690-2.

Chronic pain affects approximately one third of children and adolescents [1, 2]; many of these individuals also have co-occurring obesity [3]. These commonly co-occurring syndromes are associated with a poor quality of life compared to either disease alone [4]. Obesity is a risk factor for debilitating pain conditions in both adults and children, including osteoarthritis [5–7], migraine and chronic daily headaches [8, 9], fibromyalgia [10], and musculoskeletal pain [11]. There is support for inflammation as a potential link [12] but the molecular mechanisms of these interaction are not understood.

The obesogenic state is proinflammatory; the percentage of body mass composed of white adipose tissue rises which leads to the increase of proinflammatory cytokines such as IL-6 [13, 14]. Inflammatory pain is characterized by an increase of cytokines which bind to G protein-coupled receptors (GPCRs) and tyrosine kinase receptors to decrease the activation threshold of transient receptor potential (TRP) channels involved in pain sensation [13]. Several substances, including different classes of lipids, have been shown to have roles in pain sensation through TRP channels [15, 16]. These include oxidized lipid metabolites from linoleic acid (LA), arachidonic acid (AA), lysophospholipids and sphingolipids [17, 18]. Metabolization of AA through cyclooxygenases (COX) forms mediators like prostaglandin E_2_ which can cause inflammatory pain by binding to EP receptors, leading to subsequent activation of TRPV1 channels [19, 20]. Given the regulation of pain sensation by lipids and the modulation of lipids by obesity, there is a potential for altered lipid metabolism to underlie the association between chronic pain and obesity.

Women are affected by both chronic pain and obesity more than men [12]. Based on recent estimates, the prevalence of obesity among adolescents (12–19 years) is 22.2% and among adults (age 20 years and older) is 41.9% [21]. In the US, the prevalence of severe obesity in adults is higher in women. Pediatric pain is greater in females for most pain types, and the majority of pediatric pain patients are female [12]. Furthermore, females have a higher co-incidence of chronic pain and obesity [9, 22]. Therefore, the current study focused on female adolescents differentially affected by obesity and pain.

We are not aware of systematic studies that have examined a comprehensive array of lipids and lipid metabolites in human subjects with both chronic pain and obesity. However, there is interest in lipidomic profiling for those with obesity (e.g. [23]). Since, as described above, there are significant interactions between obesity and chronic pain in children and the potential for lipid metabolites to mediate pain, the purpose of this study was to evaluate lipid metabolomics in serum samples from a group of female adolescents without or with chronic pain and/or obesity to discover potentially modifiable lipid mediators of pain.

## Methods

Female adolescents (13–17 years, *N* = 67) were recruited for a larger trial focused on endogenous pain control. This project was approved by the Children’s Wisconsin Institutional Review Board and written consent provided by participants and parents. Recruitment of four groups was based on presence/absence of chronic pain and presence/absence of obesity as described previously [12, 24]: 1) Healthy Controls (“Control”; healthy weight and no pain; *n* = 17); 2) Chronic Pain with Healthy Weight (“Pain Non-obese”; *n* = 17); 3) Obese (no pain; *n* = 16); and 4) Chronic Pain and Obesity (“Pain Obese”; *n* = 17). The two groups with chronic pain were recruited from a multidisciplinary pain clinic at Children’s Wisconsin (formerly called Children’s Hospital of Wisconsin). The two groups without chronic pain were recruited from an outpatient clinic within the Children’s Wisconsin system and located in the same community. Participant recruitment took place from May 2018 – December 2019. Of the patients approached about the study, 17 declined (10.6%). Other than no reason given (*n* = 6), patients declined due the dislike of the blood draw (*n* = 5) or no interest in research (*n* = 4)). Details about the recruitment process are as described previously [12].

### Inclusion/exclusion criteria

Most criteria were extracted from information associated with the medical appointment visit in the electronic medical record (EMR) prior to approaching participants. Except for diagnoses, self-reported information was confirmed with the medical provider and family prior to consent discussion. Medical providers inquired directly about the use of medicinal marijuana/cannabidiol and use of illicit street drugs.

### Inclusion criteria

Female, 13–17 years old, English speaking, and, if taking psychotropic or long-acting analgesics, doses had to be stable (defined as ≥1 week of medication use). Inclusion in the groups without obesity required a BMI of 5th- < 85th percentile, based on age and gender [25]. Inclusion in the groups with obesity required a BMI ≥95th percentile, based on age and gender [25]. Subjects for the two non-pain groups answered screening questions prior to a consent discussion: 1) “Do you have any chronic illness?” Subjects who responded “yes” were excluded; 2) “Over the past three months, have you had pain?” Response options included “not at all,” “rarely,” “sometimes,” “frequently,” “all the time.” Subjects who responded “sometimes,” “frequently” or “all the time” were excluded. Self-reported days with pain (PFSD1) and worst pain intensity (PFSD4) over the past 2 weeks were used to evaluate potential between-group differences in pain characteristics [26].

### Exclusion criteria

Type 1 or Type 2 diabetes mellitus and/or metabolic syndrome, documented hypertension, cancer-related pain, sickle cell disease, and inflammatory conditions (e.g. rheumatoid arthritis, fibromyalgia, irritable bowel syndrome, celiac disease, Crohn’s disease, ulcerative colitis, lupus), or the use of metformin, Accutane, corticosteroids, asthma inhalers (daily use of the latter two within the past 2 weeks or ≥ 12 times in the past month or use within 12 hours of blood draw), medicinal marijuana/cannabidiol, immune-modulating medications, or self-reported use of illicit “street drugs”. Use of non-steroidal anti-inflammatory medications within 12 hours of blood draw. Non-English speaking.

### Lipid extraction

Samples were split into two batches (samples 1–34 and 35–67). Serum (100 μL) was aliquoted into 1.5 mL microcentrifuge tubes (Fisherbrand, Cat. No. 05408129) containing 500 μL 3:1:6 IPA:H_2_O:ethyl acetate (0.01% BHT), 10 μL Splash II Lipidomix internal standard (Avanti, Cat. No. 330709), and 10 μL Oleoyl-L-carnitine-d3 internal standard (Cayman Chemical, Cat. No. 26578). Samples were homogenized using a Qiagen TissueLyzer II (Cat. No. 9244420) at a frequency of 30 1/s for 40 s for four cycles, with a 5-minute incubation at 4 °C between each cycle. Samples were then placed in − 20 °C for 10 minutes, followed by a centrifugation at 16,300 g for 5 minutes at 4 °C. 500 μL of supernatant was transferred to new microcentrifuge tubes with a repeat centrifugation step. 450 μL of supernatant was transferred to new microcentrifuge tubes and placed in a speed vacuum. Samples were resuspended in 150 μL methanol and stored at − 80 °C. Samples were diluted 1:20 in methanol for positive mode runs and undiluted for negative mode runs. Seven MS/MS iteratives and three quality control vials were prepared per batch, per mode.

### LC/MS analysis

Extracted lipids were separated on an Agilent 1290 Infinity II LC System through a VanGuard BEH C18 Precolumn and an Acquity C18 1.7 μm column (Waters, Part No. 186002352, 2.1 × 100 mm). Injection volumes were 3 μL for positive mode and 5 μL for negative mode. A chromatography gradient was run at a flow rate of 0.500 mL/min composed of mobile phase A (ACN:H_2_O [60:40 v/v]) in 10 mM ammonium formate and 0.1% formic acid) and mobile phase B (IPA:H_2_O [90:10 v/v]) in 10 mM ammonium formate and 0.1% formic acid). The chromatography gradient started at 15% mobile phase B increasing to 30% by 2.40 min, 48% by 3.00 min, 82% by 13.20 min, and 99% by 13.80 min. Mobile phase B was then maintained at 99% until 15.40 minutes, then decreased to 15% by 16.00 minutes and held at 15% to 20.00 minutes.

Untargeted lipidomics analyses were performed in both positive and negative mode on an Agilent 6546 quadrupole time-of-flight MS dual AJS ESI mass spectrometer connected to the LC system (QTOF-LC/MS). For positive mode, the source gas temperature was set to 250 °C, with a gas flow of 12 L/min, nebulizer pressure of 35 psig, sheath gas temperature of 300 °C and sheath gas flow of 11 L/min. Reference masses used for positive mode were 121.050873 and 922.009798 m/z. For negative mode, the source gas temperature was set to 250 °C, with a gas flow of 12 L/min, nebulizer pressure of 30 psig, sheath gas temperature of 375 °C and sheath gas flow of 12 L/min. Reference masses for negative mode were 112.9855 m/z, 966.0007 m/z, and 1033.9881 m/z. In both modes, VCap voltage was set at 4000 V, fragmentor at 190 V, skimmer at 75 V and Octopole RF peak at 750 V.

Samples were acquired in the mass scan range of 119–1500 m/z (positive) and 100–1500 m/z (negative) at a rate of 4 spectra/sec. Tandem mass spectrometry was performed with a fixed collision energy of 25.00 V at a scan rate of 2 spectra/sec.

### Data processing

Raw data from LC-MS experiments was evaluated using Agilent MassHunter Workstation and stored in .d format. MS/MS data was analyzed using Agilent MassHunter Qualitative Analysis and Lipid Annotator for lipid identification. Lipids identified in Lipid Annotator with a mass accuracy of < 0.05 ppm were exported in PCDL format to create comprehensive libraries. Lipid identifications were checked for accuracy through retention time correlations with lipids of the same class and by assessing fragmentation patterns. Lipid annotations were further screened for redundancies using the following criteria: identifications for the same lipid were filtered by removing any duplicates if the retention time difference was < 0.10 min or if the lower intensity annotation was < 25% of the more abundant one. To determine total unique identifications, positive and negative mode data were compared for identical lipids annotated in both modes and the lower abundance lipids were excluded. Compounds in each sample were analyzed and integrated based on the generated library from batch 1 (batch 2 had no unique annotations) using Agilent Profinder Analysis through Targeted Feature Extraction, and peak heights were exported to .csv files for further analysis using an in-house R script [27]. Pathway analysis of dataset was performed with lipid ontology (LION) [28].

### R data curation

Data curation was performed in R (version 4.1.1) using an in-house script [27]. Any lipid abundance in samples less than the corresponding processed blank abundance was removed. Lipid variables that were not present in at least 30 of the samples were removed and a lipid correlation was run to identify and delete duplicate lipid annotations. Standards were then plotted to assess variation between samples and saved as PDF files. Lipids identified in positive and negative mode were combined, and the lipid with the lower abundance was dropped. Lipids normalized to their respective internal standard. In positive mode, the internal standard for CE was highly variable and missing from some samples which led to its removal. In negative mode, the internal standard for SM, GM3, HexCer, PA, and PS were not detected and were removed. Data was exported as .csv files.

### Other statistical analyses

Demographic data were analyzed by non-parametric Kruskal-Wallis one-way analysis of variance (ANOVA), exact Mann Whitney tests for pairwise comparisons, and Fisher-Freeman-Halton’s exact test (SPSS v26, IBM Corporation, Somers, NY, and Cytel StatXact v8, Cambridge, MA). No adjustments were made for multiple comparisons. *P* < 0.05 was considered significant; unadjusted values are given. Data are presented as mean (standard deviation) or median (25–75%).

## Results

Table 1 shows the demographic and anthropometric data for the four groups of subjects. There were no differences in the age (*P* > 0.32), race (*P* = 0.23), nor ethnicity (*P* > 0.60) between the four groups. As expected, BMI was greater in the Obese and Pain Obese groups compared to the Control and Pain Non-obese groups. Furthermore, as expected, there were no differences between the two non-obese groups (Control and Pain Non-obese) nor between the two obese groups (Obese and Pain Obese). Self-reported days with pain over the past 2 weeks (PFSD1) and worst pain intensity over the past 2 weeks (PFSD4) were greater in the Pain Non-obese and Pain Obese groups compared to the Control and Obese groups, but not different within the no chronic pain groups or within the chronic pain groups. Pain location is summarized to best represent between group differences. No differences were found between groups (*P* > 0.06). Most participants in both chronic pain groups presented with headache/migraine pain, followed by extremity pain (Pain Non-obese group), and abdominal pain (Pain Obese). “Other” pain conditions were combined into a single category (included back pain and joint pain).

**Table 1:** Demographic and anthropomorphic data

	Healthy Controls	Pain/Healthy Weight	Obese Alone	Pain & Obese	*P*-value
**N**	17	17	16	17	
**Age (years)**	14.8 [1.3]	15.5 [1.2]	14.7 [1.6]	15.1 [1.4]	> 0.32
**Race**					0.23
**White**	15	16	12	11	
**African-American**	0	1	0	2	
**Native American**	0	0	0	1	
**More than 1**	2	0	4	3	
**Hispanic**					> 0.60
**No**	16	13	13	14	
**Yes**	1	3	3	3	
**No answer**	0	1	0	0	
**BMI (percentile)**	55 [41–70]	54 [38–73]	97 [95–98]*	98 [96–99]*	< 0.001
**Pain Location (*n*, %)**					**>** 0.06
Headache/Migraine		10 (58.8)		15 (88.2)	
Extremities		3 (17.6)		0	
Abdomen		1 (5.9)		2 (11.8)	
Other		3 (17.6)		0 (0.0)	
**Pain Duration (months)**		24 [18–30]		24 [8–60]	> 0.18
**PFSD1 (days)**	1.0 [0.0–2.0]	13.0 [5.5–14.0]**	1.0 [0.0–3.0]	7.0 [3.5–13.0]**	< 0.001
**PFSD4 (worst pain intensity)**					< 0.001
	0.0 [0.0–5.0]	8.0 [7.0–9.5]**	0.0 [0.0–6.0]	8.0 [5.0–8.0]**	

Data are shown as mean [SD] or median [25th–75th %] where appropriate

*BMI* Body mass index, *PFSD1* Pain Frequency Severity Duration Scale-1 (PFSD1; days with pain in the past 2 weeks), Pain Frequency Severity Duration Scale-4 (PFSD4; worst pain intensity in the past 2 weeks)

*different from Healthy Controls and Pain/Healthy Weight groups (*P* < 0.001)

**different from Healthy Controls and Obese Alone groups (*P* range, 0.003 to < 0.001)

### Individuals with chronic pain and healthy weight have a lipid signature matching those with obesity alone and individuals with chronic pain and obesity

**Fig. 1:**
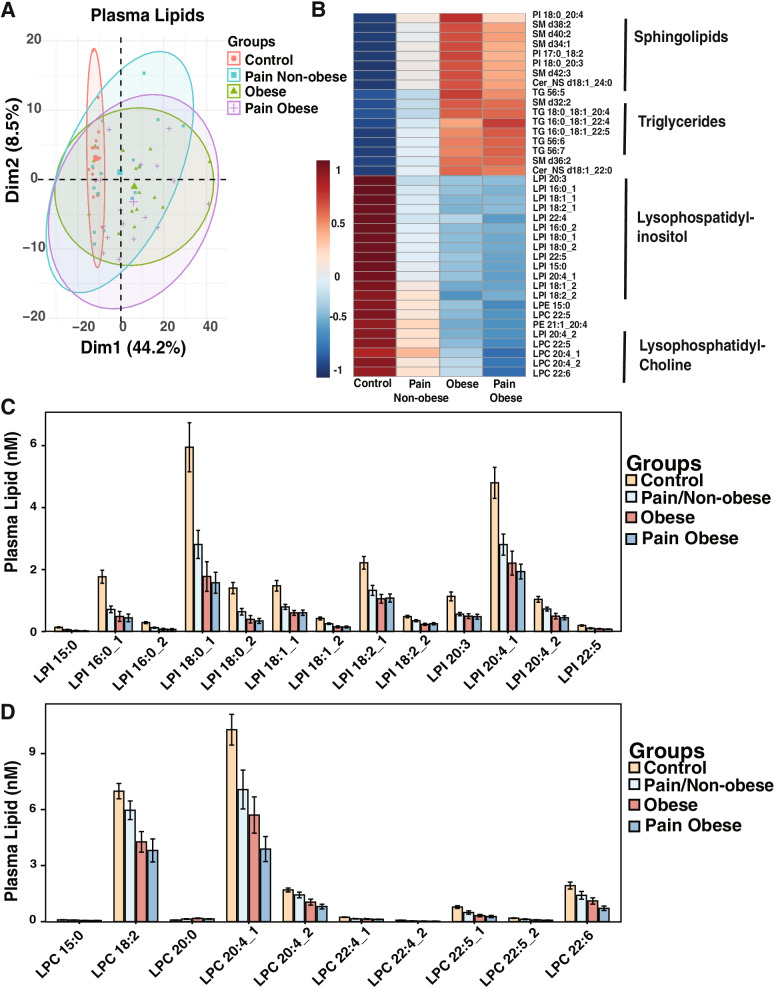
Healthy weight individuals with chronic pain have serum lipid profiles that match the obese and chronic pain obese groups. **A** Principal component analysis between serum of control (*n*=17), obese (*n*=16), pain non-obese (*n*=17), and pain obese (*n*=17) individuals. **B** Heat map of group averages showing lipid class distribution between control, obese, pain non-obese, and pain obese groups. **C** Lysophosphatidylinositol (LPI) species abundance in serum between control, obese, pain non-obese, and pain obese individuals. **D** Lysophosphatidylcholine (LPC) species abundance in plasma between control, obese, pain non-obese, and pain obese groups. *n*=16-17 per group, Data are presented as means ± SEM.

Due to the high co-occurrence of chronic pain and obesity, we wanted to assess lipid changes in the serum that could serve as identifiable biomarkers of chronic pain. To measure serum lipids, we performed global lipidomics using QTOF-LC/MS. Principal component analysis (PCA) on the four study groups found that the Control group had distinct lipid clustering while there was significant overlap in lipids shared between the three clinical groups (Fig. 1A). There were 426 lipids detected in at least one sample, while 383 lipids were detected in all samples. Of the 383 lipids detected in all samples, there were 234 lipids observed to be significantly different between the four groups, whereas 149 lipids were not significantly different between the four groups (Supplementary Fig. S1A & B).

The lipids that had the greatest variability between groups included lysophospholipids such as lysophosphatidylinositol (LPI), lysophosphatidylcholine (LPC), and lysophosphatidyethanolamine (LPE) as well as sphingolipids and triglycerides (Fig. 1B). The Pain Non-obese group had a similar lipid profile to the Obese and Pain Obese groups with lower levels of LPI and LPE when compared to the Control group (Figs. 1C & S1C). Interestingly, one of the LPE species that was higher in the Control group included LPE 15:0; odd chain fatty acids like these arise from the diet, gut microbiota, or peroxisomal lipid processing but cannot be synthesized de novo [29]*.* The LPC differences between groups displayed acyl-chain specificity, with the Pain Non-obese group having elevated LPC 18:2 that was similar to control, but lower levels of LPC 20:4 and LPC 22:6 closer to the Obese and Pain Obese groups. As would be expected, both groups with obesity had elevated levels of serum triglycerides compared to groups with healthy weight independent of chronic pain (Figs. 1B & S1D). Both groups with pain had elevated triglycerides that contained polyunsaturated fatty acids (PUFAs), with the Pain Non-obese group having levels lower than those in the Pain Obese group (Fig. S1D).

### Individuals with chronic pain and healthy weight have an elevation in sphingolipids but a decrease in lysophospholipids

**Fig. 2:**
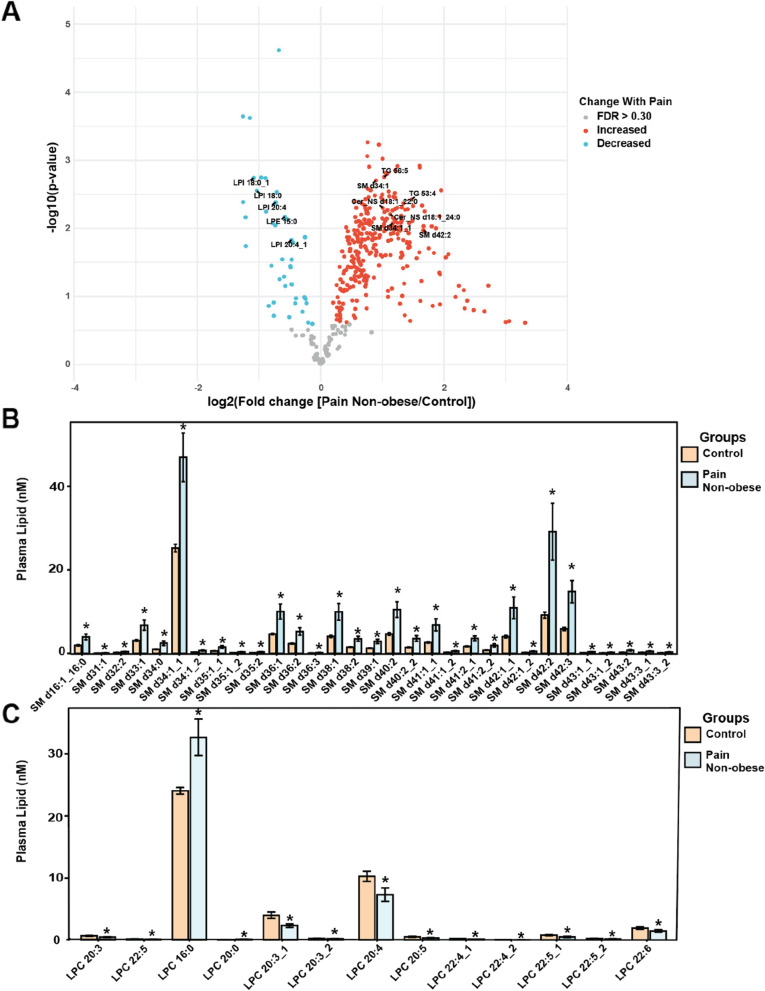
Healthy weight individuals with chronic pain have decreased lysophospholipid and increased ceramide levels compared to control. **A** Volcano plot illustrating significant and high fold change between plasma of control and pain non-obese individuals. FDR q > 0.30 red dots indicate significant increase, FDR q < 0.30 blue dots indicate significant decrease. **B** Sphingomyelin (SM) species abundance in plasma significant between control and pain non-obese individuals. **C** Lysophosphatidylcholine (LPC) species abundance in plasma significant between control and pain non-obese individuals. *n*=17 per group, Data are presented as means ± SEM. *p < 0.05

Although 234 lipids were found to be statistically different when considering all 4 groups, the Control group was the driving force of this finding. Tukey posthoc analysis showed distinct lipid changes between each of the four groups, with a lipid signature emerging in the Pain Non-obese group compared to the Control. Comparing the fold change between Pain Non-obese and Control groups showed a decrease in lysophospholipids LPI and LPE but an increase in sphingolipids such as ceramides and sphingomyelin (Fig. 2A). Several sphingomyelin species were elevated in the Pain Non-obese group, with the most abudant being SM d34:1 (45.6 ± 23.2 [SD] nM), SM d42:2 (27.5 ± 27.3 nM), and SM d42:3 (14.2 ± 10.6 nM) (Fig. 2B). Some ceramide species also demonstrated a signficant increase, with the most abudant species being Cer_NS d18:1_24:0 (1.7 ± 1.1 nM (Supplementary Fig. S2A). Lysophospholipids were decreased in the Pain Non-obese group compared to the Control, with a broad range of LPIs showing decreases (Fig. S2B). Long chain and very long chain LPC species were also lower in the Pain Non-obese group, with the only exception being LPC 16:0. Notably, this LPC has the highest abudance for both Control and Pain Non-obese groups at 24 ± 2.2 nM and 32.7 ± 11.8 nM, respectively (Fig. 2C).

### Lipid changes associated with chronic pain and healthy weight are exacerbated in individuals with chronic pain and obesity

**Fig. 3:**
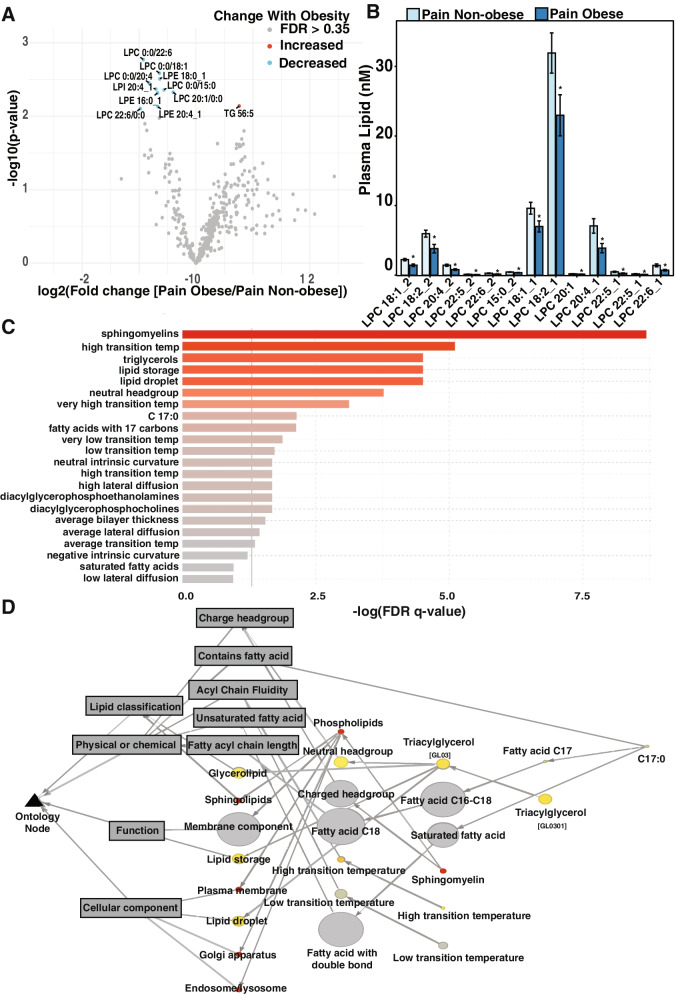
Obesity exacerbates the chronic pain signature decreasing LPC. **A** Volcano plot illustrating significant and high fold change between plasma of pain non-obese and pain obese groups. FDR q > 0.35 red dots indicate significant increase, FDR q < 0.35 blue dots indicate significant decrease. **B** Lysophosphatidylcholine (LPC) species abundance in plasma significant between pain lean and pain obese individuals. **C** Lipid ontology (LION) analysis (**D**) and pathway assessment comparing plasma of pain non-obese and obese groups to control and obese individuals. *n*=17 per group, Data are presented as means ± SEM. *p < 0.05

The Pain Non-obese group had decreases in lysophospholipids LPI, LPE, and LPC when compared to the Control group (Fig. 2). These lysophospholipids were further decreased in those with chronic pain and obesity when compared to those with chronic pain and a healthy weight (Fig. 3A). Several LPC species were significantly decreased in the Pain Obese group compared to those in the Pain Non-obese group, with the most abundant being LPC 18:2, LPC 18:1, and LPC 20:4 (Fig. 3B). Low abundant but signficant LPC differences included odd chain LPC 15:0. LPE and LPI showed a similar decrease in these phospholipids in the Pain Obese group. Specifically, there were significant decreases in four phospholipids: LPE 15:0, LPE 16:0_1, LPE 18:0_1 and LPE 20:4_1. Notably, abundance of each LPE increased with acyl chain length. LPI 18:1_1 and LPI 20:4_1 were the only two LPI species with a significant decrease in the Pain Obese group when compared to the Pain Non-Obese group, with the more abundant LPI being 20:4_1 (Supplementary Fig. S3A & B). Other differences between the groups were clearly driven by accumulation of stored lipids in obesity such as increased triglycerides in those with chronic pain and obesity compared to those with chronic pain and a healthy weight. (Supplementary Fig. S3C).

To investigate possible pathways associated with pain independent of obesity, we used lipid ontology (LION) analysis comparing the groups with chronic pain to the groups with no pain independent of body weight changes [28]. Sphingomyelins and triacylglycerols were ranked highest, suggesting their involvement in chronic pain (Fig. 3C). Functional pathway assessment further demonstrated sphingomyelin’s pain association and found an association with vesicles formation (Fig. 3D). Association with vesicle formation also reflects changes in LPI, LPE, and LPC which contribute to the high degree of curvature in vesicles.

## Discussion

A large proportion of youth in the US suffer from chronic pain that can be debilitating. The highest predictors for adult chronic pain are the occurrence of adolescent obesity and chronic pain [30]. Little is known about the etiology of this phenomenon despite the deleterious impact. Our goal was to understand the molecular interplay between chronic pain and obesity in adolescents by performing global lipidomics on serum samples. Key findings include the identification of changes in the lipid profile associated with chronic pain, and the finding that some of these changes are exacerbated when chronic pain co-occurs with obesity.

Specifically, we demonstrate that individuals with chronic pain and healthy weight had lipid profiles similar to those with obesity alone, and those with chronic pain and obesity. Lipids with the greatest variability between all four groups included sphingolipids, triglycerides, and lysophospholipids. The Pain Non-obese group demonstrated a significant elevation in sphingolipids like ceramides and several sphingomyelin species when compared to Controls. Those with chronic pain and healthy weight also had lower levels of LPI, LPC, and LPE when compared to Controls, but similar abundances as those with obesity and chronic pain with obesity. Notably, some lipid changes associated with chronic pain were exacerbated in the obese state with LPI, LPE, and LPC species being further decreased in the Pain Obese group. Pathway analysis ranked sphingomyelins and triacylglyerols as having the highest association with pain, and functional clustering identified lipids that regulate vesicle formation and lipid droplets (Fig. 3). The changes in numerous species in a class, rather than individual lipids suggests that there may be altered production of lysophospholipids and sphingolipids in chronic pain. Further studies are needed to determine if these lysophospholipids and sphingolipids have a functional role in the etiology of chronic pain, and a role in the link between chronic pain and obesity.

As indicated, the two major lipid classes associated with chronic pain were sphingolipids and lysophospholipids. Individuals in the Pain Non-obese group demonstrated higher levels of several sphingolipid species compared to Controls, and pathway analysis ranked sphingomyelins as having the highest association with pain, regardless of weight. Sphingolipids such as sphingomyelin and ceramides are a major component of the central nervous system, where they are abundant in the plasma membrane of myelin sheaths and serve as possible biomarkers for the demyelination that occurs in multiple sclerosis [31, 32]. Several sphingolipid species elevated in patients with multiple sclerosis include, but are not limited to: Cer 16:0, Cer 18:1, Cer 24:0, SM 20:0, SM 22:0, SM 24:0, SM 24:1 [33, 34]. We similarly observed increases in Ceramide species contianing 16:0, 18:1, 24:0, 20:0, and 22:0 acyl chains in chronic pain with or without obesity.

We also observed decreases in LPI, LPE, and LPC species that were exacerbated in those with chronic pain and obesity. LPC and LPE can be processed into lysophosphatidic acid (LPA), and its presence in the spinal cord serves to resolve inflammation and possibly assists with remyelination. In patients with multiple sclerosis, LPAs were found to be reduced in the serum [35]. However, LPA and its receptor has also demonstrated a signalling role in the initiation of neuropathic pain and fibromyalgia [36, 37]. Both chronic pain groups showed a significant decrease in several LPC and LPE species, which could result in a decrease in LPA and thus contribute to pain. Despite these findings suggesting a role for sphingolipids, LPI, LPE, and LPC in chronic pain, further research is needed to understand their production and signalling function in those with chronic pain and obesity.

One of the leading mechanisms connecting the comorbidities of chronic pain and obesity is inflammation [12]. Several of the lipids that are altered in chronic pain are known to regulate inflammation such as lysophospholipids, polyunsaturated fatty acids, and sphingolipids. LPC 16:0 has been shown to induce inflammation and in our study LPC 16:0 was the most abundant LPC in the serum as well as the only LPC increased in those with chronic pain [38]. We observed other LPCs to be decreased in chronic pain including LPCs containing docosahexaenoic acid (DHA, 22:6) acyl chain which have been shown to be anti-inflammatory and capable of mitigating the pro-inflammatory actions of LPC 16:0 [39]. Notably, this decrease in LPCs was exacerbated in the Pain Obese group. We also observed other anti-inflammatory lysophospholipids, such as LPI species, to be decreased in both chronic pain groups but lowest in the Pain Obese group. Treatment of macrophages with LPI is known to decrease inflammatory cytokines such as IL-6 and IL-1β, and induction of inflammation by LPS decreases LPI abundance [40]. Ceramides have been shown to increase inflammation through activation of NOD-like receptor pyrin domain containing 3 (NLRP3) to increase the production of proinflammatory cytokines IL-1β and IL-18 [41, 42]. Our data show increased ceramides in chronic pain and obesity which aligns with a proinflammatory state, but the tissue where these ceramides are produced and their impact on local inflammation is unknown. Whether the inflammation is preceding the lipid changes, or if the lysophospholipids and sphingolipids are directly signaling for an inflammatory response that would exacerbate the chronic pain state is also unknown.

Several lipid species that were significantly different between groups with chronic pain vs no pain contained an odd number of carbons in the acyl chain, such as C15 or C17. In mammals, synthesis of fatty acids by the fatty acid synthase complex occurs in two carbon units meaning that only even acyl chains are produced by de novo lipid synthesis, whereas odd chain fatty acids such as C15 and C17 carbon chains typically come from dietary origins or are produced by the gut microbiota. In mice on HFD, C15 and C17 fatty acids are decreased and correlated with lower abundance of several gut microbial species including *Lactobacillus, Bifidobacterium, and Akkermansia* that are known to produce these odd chain fatty acids [43]. However, other studies have shown that in humans C15 and C17 lipids are independently derived from dietary intake or α-oxidation in the persoxisome, respectively [44]. We did not determine the source of the C15 and C17 lipids in our samples, but the difference could be due to either changes in gut microbiota, diet, or altered peroxisomal lipid processing. Altered gut microbiota have been associated with fibromyalgia and chronic back pain [29]. Despite these promising results, few studies have explored the association of chronic pain and gut microbiota in adolescent populations [29]. Similarly, diets low in omega-3 fatty acids, vitamin B12, and vitamin D have been associated with chronic pain, but few studies have explored the link in adolescents beyond disordered eating [45–47]. More work is needed to explore the importance of gut microbiota, diet, and α- oxidation in adolescent pain and their association with obesity.

One challenge in the current study is the distinction of isomers in the lipidomics method which led to duplicate annotations distinguished by number such as LPI 16:0_1 and LPI 16:0_2. These repeated annotations represent regioisomers labeled 1 and 2 in which the acyl chain is attached at the sn-1 or sn-2 position. The hydrophobic interaction of the c18 column is stronger when acyl chains are attached in the sn-1 position leading to a distinct retention time, and fragmentation pattern in positive mode due to water loss when the acyl chain is in the sn-2 position [48, 49]. Others have shown acyl chain specificity for each position, with sn1 position being occupied primarily by saturated lipids while sn2 favors PUFAs. These data suggest that there may be functional specificity for regioisomers and their regulation that requires further exploration. Our current method of extraction is not designed to prevent the intra-molecular acyl chain migration which prevents absolute quantification of each regioisomer and prevents exploration of the functional role [50]. Moreover, despite the distinct regioisomers, all are decreased in chronic pain, obesity, and those with chronic pain and obesity suggesting that they may be a processing derivative. Future work should use direct sample collection and isolation to allow absolute quantification of regioisomers in disease states, specifically in chronic pain and obesity [50–52].

Overall, these results demonstrate that there is a lipid signature associated with chronic pain in female adolescents, and that it is exacerbated with the addition of an obese state. The major lipid classes associated with chronic pain include lysophospholids and sphingolipids that have a role as signaling molecules known to regulate the perception of pain and mediate inflammation. Other interesting features emerged including the presence of exogenously derived acyl chains that have an odd carbon number, suggesting a further need to investigate the gut-brain axis in adolescents with chronic pain. Future work should assess targeted runs, collection of fecal samples, and dietary questionaires to probe these outstanding questions. The ultimate goal is to apply these novel lipidomic findings to develop new approached to mitigate pain in adolescents with obesity.

## Supplementary Information

**Additional file 1: Supplemental Fig. 1.** A) Analysis of variance (ANOVA) between plasma of control (*n* = 17), obese (*n* = 16), pain lean (*n* = 17), and pain obese (*n* = 17) individuals. Red dots indicate significant lipids (*p* < 0.05). B) Venn diagram showing overlap of lipid species in plasma between control, obese, pain non-obese, and pain obese groups. C) Lysophosphatidylethanolamine (LPE) species abundance in plasma between control, obese, pain non-obese, and pain obese groups. D) Triglyceride (TG) species abundance in plasma between control, obese, pain non-obese, and pain obese groups. *n*=16-17 per group, Data are presented as means ± SEM. *p < 0.05. **Supplemental Fig. 2.** A) Ceramide (Cer) species abundance in plasma significant between control and pain non-obese individuals. B) Lysophosphatidylinositol (LPI) species abundance in plasma significant between control and pain non-obese groups. *n* = 17 per group, Data are presented as means ± SEM. *p < 0.05. **Supplemental Fig. 3.** A) Lysophosphatidylethanolamine (LPE) species abundance in plasma between pain non-obese and pain obese groups. B) Lysophosphatidylinositol (LPI) species abundance in plasma significant between pain non-obese and pain obese groups. C) Triglyceride (TG) species abundance in plasma between pain non-obese and pain obese groups. *n* = 17 per group, Data are presented as means ± SEM. *p < 0.05.

## Data Availability

The datasets used and/or analyzed during the current study are available from the corresponding author on reasonable request.
