# Supplementary material for: Lipid signatures of chronic pain in female adolescents with and without obesity
Source: Lipids Health Dis. 2022 Aug 30;21:80. doi: 10.1186/s12944-022-01690-2 (PMC9426222; doi:10.1186/s12944-022-01690-2)

Supplemental Figure Legends:

Supplemental Figure 1 A) Analysis of variance (ANOVA) between plasma of control (n = 17), obese (n = 16), pain lean (n = 17), and pain obese (n = 17) individuals. Red dots indicate significant lipids ($p < 0.05$). B) Venn diagram showing overlap of lipid species in plasma between control, obese, pain non-obese, and pain obese groups. C) Lysophosphatidylethanolamine (LPE) species abundance in plasma between control, obese, pain non-obese, and pain obese groups. D) Triglyceride (TG) species abundance in plasma between control, obese, pain non-obese, and pain obese groups. n=16-17 per group, Data are presented as means ± SEM. *$p < 0.05$

Supplemental Figure 2 A) Ceramide (Cer) species abundance in plasma significant between control and pain non-obese individuals. B) Lysophosphatidylinositol (LPI) species abundance in plasma significant between control and pain non-obese groups. n=17 per group, Data are presented as means ± SEM. *$p < 0.05$

Supplemental Figure 3 A) Lysophosphatidylethanolamine (LPE) species abundance in plasma between pain non-obese and pain obese groups. B) Lysophosphatidylinositol (LPI) species abundance in plasma significant between pain non-obese and pain obese groups. C) Triglyceride (TG) species abundance in plasma between pain non-obese and pain obese groups. n=17 per group, Data are presented as means ± SEM. *$p < 0.05$

Supplemental Figure 1

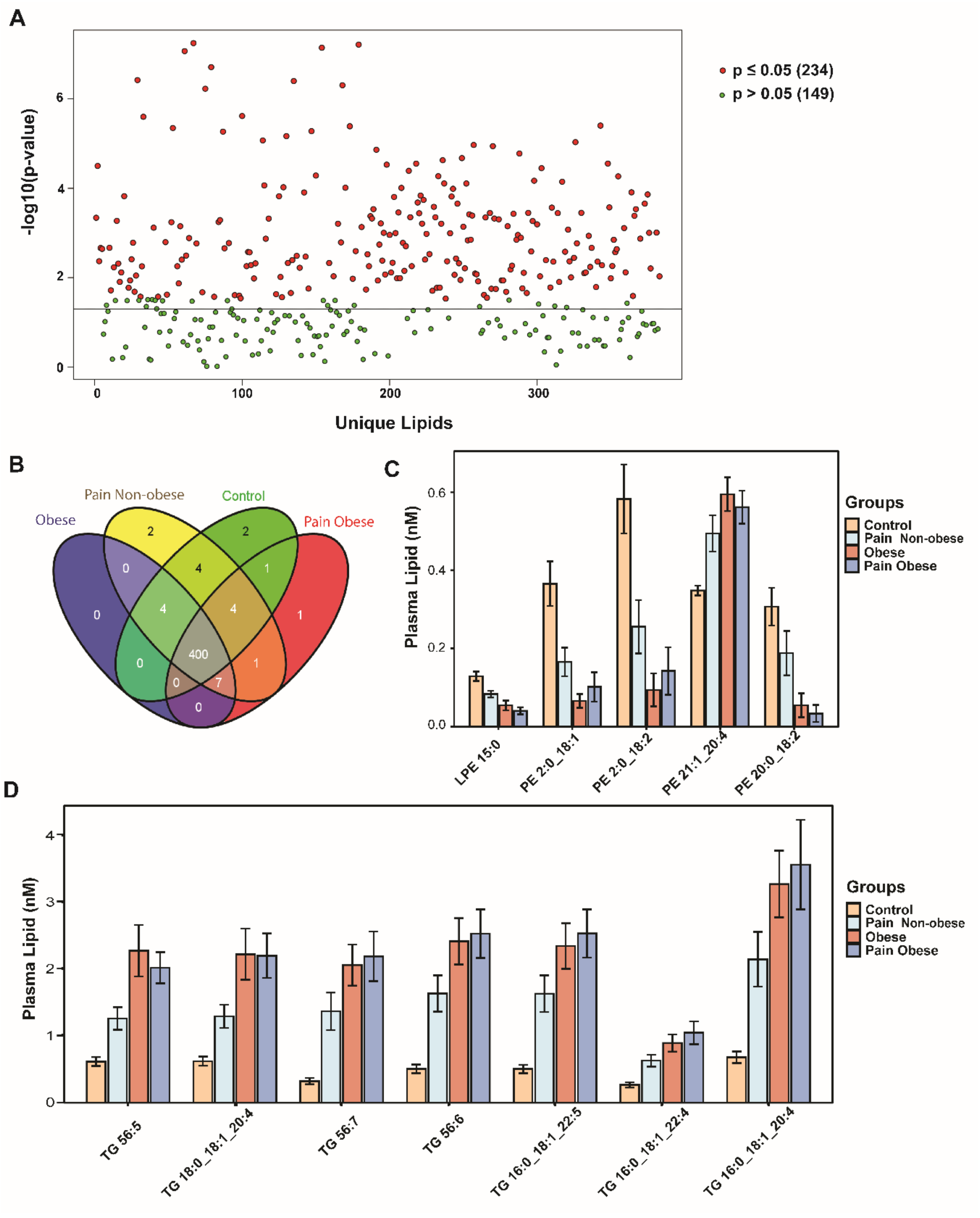

Supplemental Figure 2

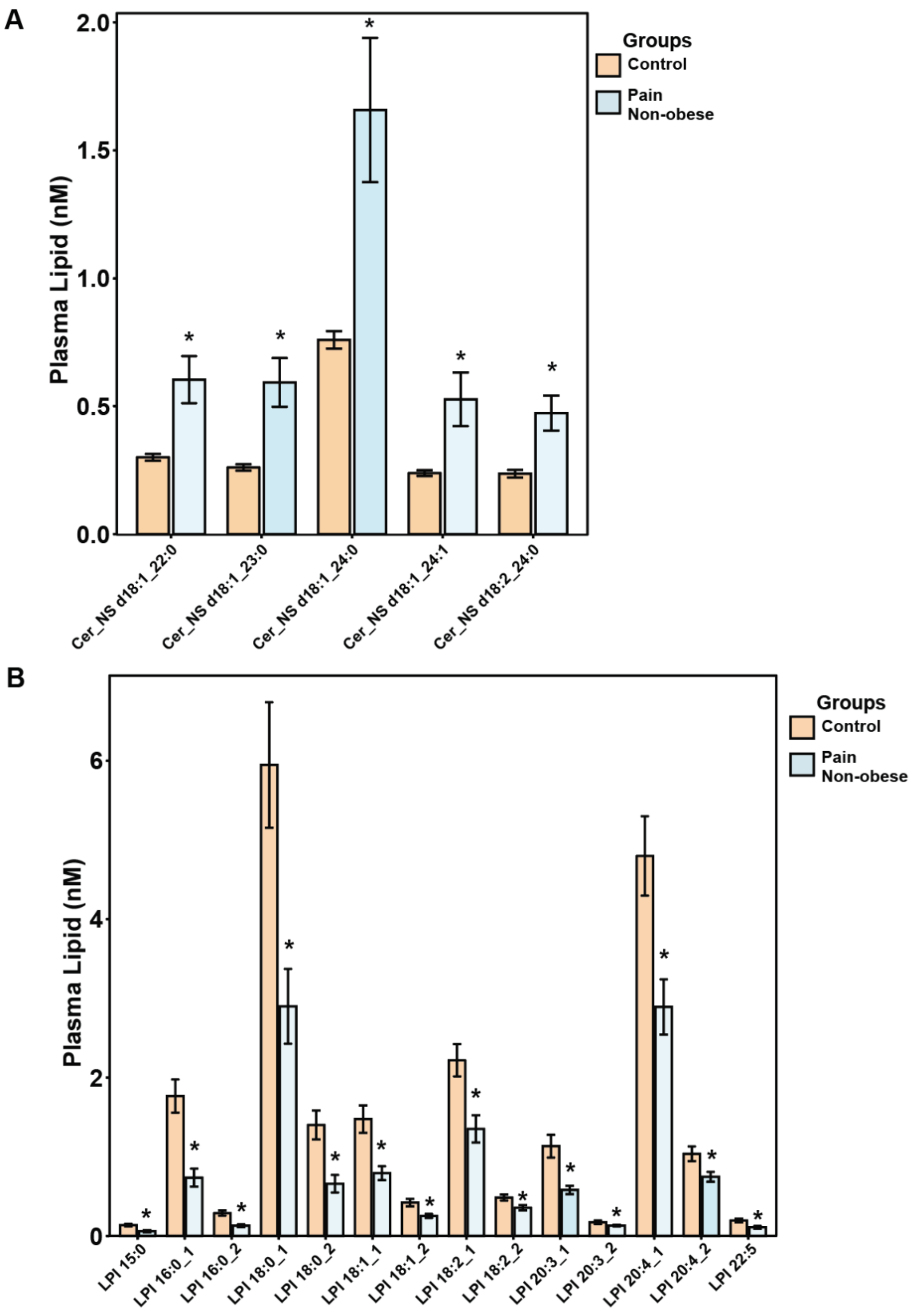

Supplemental Figure 3

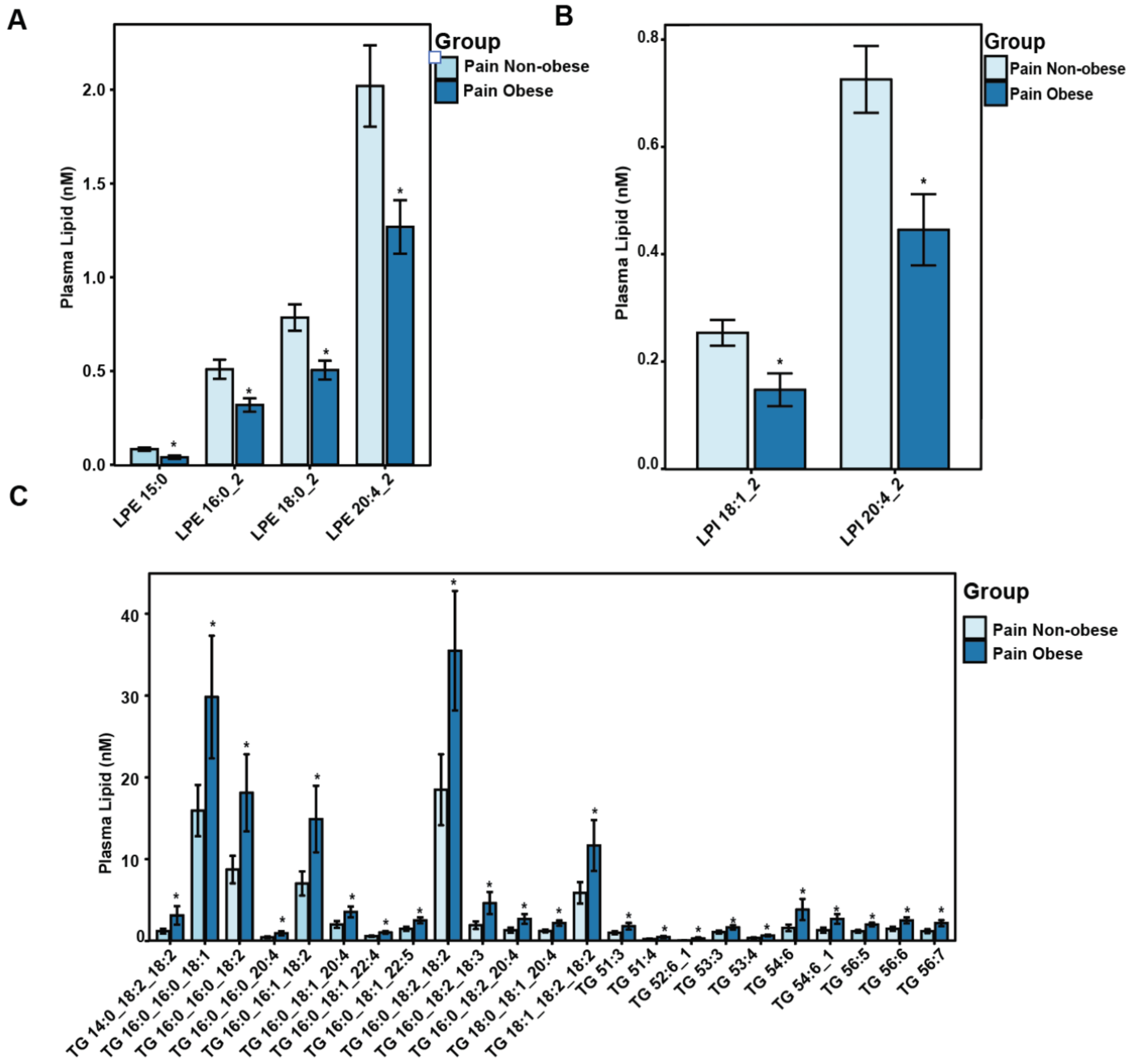

Supplement: Supplementary file 1 — Additional file 1: Supplemental Fig. 1. A) Analysis of variance (ANOVA) between plasma of control (n = 17), obese (n = 16), pain lean (n = 17), and pain obese (n = 17) individuals. Red dots indicate significant lipids (p < 0.05). B) Venn diagram showing overlap of lipid species in plasma between control, obese, pain non-obese, and pain obese groups. C) Lysophosphatidylethanolamine (LPE) species abundance in plasma between control, obese, pain non-obese, and pain obese groups. D) Triglyceride (TG) species abundance in plasma between control, obese, pain non-obese, and pain obese groups. n=16-17 per group, Data are presented as means ± SEM. *p < 0.05. Supplemental Fig. 2. A) Ceramide (Cer) species abundance in plasma significant between control and pain non-obese individuals. B) Lysophosphatidylinositol (LPI) species abundance in plasma significant between control and pain non-obese groups. n = 17 per group, Data are presented as means ± SEM. *p < 0.05. Supplemental Fig. 3. A) Lysophosphatidylethanolamine (LPE) species abundance in plasma between pain non-obese and pain obese groups. B) Lysophosphatidylinositol (LPI) species abundance in plasma significant between pain non-obese and pain obese groups. C) Triglyceride (TG) species abundance in plasma between pain non-obese and pain obese groups. n = 17 per group, Data are presented as means ± SEM. *p < 0.05. [file 12944_2022_1690_MOESM1_ESM.pdf]
